# Antimicrobial Properties of the Ag, Cu Nanoparticle System

**DOI:** 10.3390/biology10020137

**Published:** 2021-02-10

**Authors:** Xinzhen Fan, L’Hocine Yahia, Edward Sacher

**Affiliations:** 1 Laboratoire d’Innovation et d’Analyse de Bioperformance, Département de Génie Mécanique, Polytechnique Montréal, CP 6079, Succursale C-V, Montréal, QC H3C 3A7, Canada; Xinzhen.fan@polymtl.ca (X.F.); lhocine.yahia@polymt.ca (L.Y.); 2 Département de Génie Physique, Polytechnique Montréal, CP 6079, Succursale C-V, Montréal, QC H3C 3A7, Canada

**Keywords:** antibacterial, biofilm, metal nanoparticles

## Abstract

**Simple Summary:**

The antimicrobial properties of Ag and Cu nanoparticles, their mixtures and their alloys, are reviewed.

**Abstract:**

Microbes, including bacteria and fungi, easily form stable biofilms on many surfaces. Such biofilms have high resistance to antibiotics, and cause nosocomial and postoperative infections. The antimicrobial and antiviral behaviors of Ag and Cu nanoparticles (NPs) are well known, and possible mechanisms for their actions, such as released ions, reactive oxygen species (ROS), contact killing, the immunostimulatory effect, and others have been proposed. Ag and Cu NPs, and their derivative NPs, have different antimicrobial capacities and cytotoxicities. Factors, such as size, shape and surface treatment, influence their antimicrobial activities. The biomedical application of antimicrobial Ag and Cu NPs involves coating onto substrates, including textiles, polymers, ceramics, and metals. Because Ag and Cu are immiscible, synthetic AgCu nanoalloys have different microstructures, which impact their antimicrobial effects. When mixed, the combination of Ag and Cu NPs act synergistically, offering substantially enhanced antimicrobial behavior. However, when alloyed in Ag–Cu NPs, the antimicrobial behavior is even more enhanced. The reason for this enhancement is unclear. Here, we discuss these results and the possible behavior mechanisms that underlie them.

## 1. Introduction

As the antibiotic resistance of microbes to drugs grows, nanotechnology provides us an opportunity to resolve this problem [1,2]. Metal NPs, referred to as nanobiotics, have been proposed as novel antimicrobial agents. They have the potential to reduce or eliminate the continuous emergence of bacterial resistance [3]. The metals used for these NPs are almost exclusively heavy metals, such as Ag and Cu.

**Figure 1:**
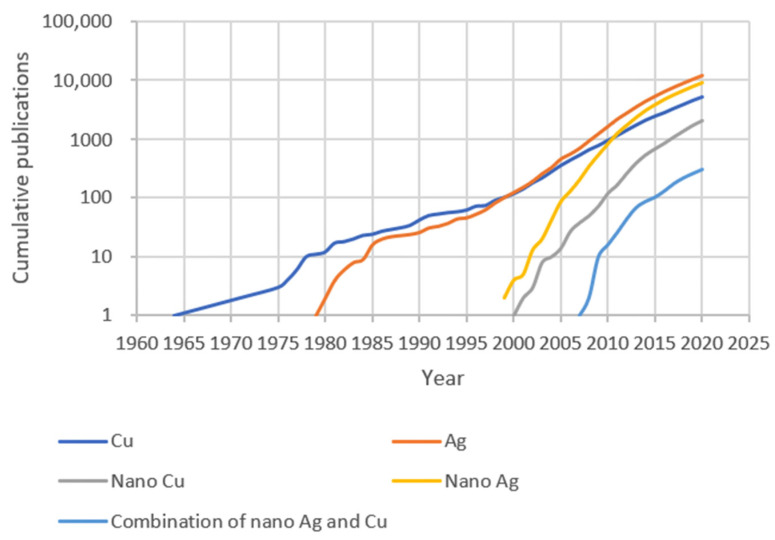
Cumulative numbers of publications on antimicrobial Cu and Ag, as of 1 December 2020. The bibliometric data were searched in the Polytechnique Montréal library Compendex database, as indicated in the Appendix A.

Ag has been utilized as an antimicrobial agent for several millennia, since Hippocrates prescribed the use of Ag to treat ulcers [4,5]. As nanotechnology has developed [6], Ag NPs have become widely used in antimicrobial applications, especially in combatting antibiotic-resistant bacteria and nosocomial infections [5]. As for Cu, its antiseptic potential was recorded four thousand years ago [7]. Indeed, the first report of Cu as an antimicrobial agent predates that of Ag. Despite this, antimicrobial studies of Ag are more common than those of Cu. What is noteworthy is that, compared to the large number of antimicrobial studies of Ag or Cu, the number of the publications involved in the combination of antimicrobial Ag and Cu nanomaterials is only approximately 300, so far (Figure 1).

Despite this, their exact antimicrobial mechanisms are still elusive. Currently proposed theories all have limitation, and cannot explain the antimicrobial activities in all situations. Recently, AgCu nanoalloys were reported to have antimicrobial properties far greater than either Ag or Cu NPs [8,9]. As it is the NP surface that participates in all the proposed mechanisms, it is our position that the physicochemical surface characterization of NPs, particularly their surfaces, will determine the actual reason(s) behind antimicrobial behavior. It is our purpose to discuss and summarize the antimicrobial activities of Ag and Cu NPs, their combinations and alloys.

## 2. Biofilm Contamination

### 2.1. Bacteria and Fungi

The majority of bacteria can be classified into two types: Gram-positive and Gram-negative. The main difference between such bacteria is their cell structures: Gram-positive bacteria have a thick layer of peptidoglycan in the cell walls, while the peptidoglycan layer of Gram-negative bacteria is thinner, and covered with another lipid membrane. Mostly, *Staphylococcus aureus* is used to represent Gram-positive bacteria, and *Escherichia coli*, Gram-negative bacteria, in antibacterial experiments. Some researchers have found Gram-positive bacteria to be more sensitive to NPs, because they consider the cell wall structure of Gram-negative bacteria to be more complex [10,11]. In contrast, other researchers believe that Gram-negative bacteria are more susceptible to antibacterial Ag NPs, as it is easier for Ag ions to penetrate the thinner cell walls of Gram-negative bacteria [12,13].

Contamination by fungi has also become a significant healthcare concern. Due to the presence of fungal eukaryotic cells, infections caused by fungi are more difficult to diagnose and treat than those caused by bacteria [14]. The most common fungus, *Candida albicans* [15], can survive, proliferate and spread for several weeks, on either dry or wet surfaces, and may cause bloodstream infections that have a high mortality rate. It has been reported that Ag NPs can inhibit the growth of fungal strains, and further damage fungus cells [16,17,18]. By contrast, Cu NPs exhibit favorable antifungal efficiency mainly in the field of fungus-induced plant diseases, rather than of human diseases [19,20,21]. However, Ag, Cu, and AgCu nanoalloy NPs cannot inhibit and kill *Candida albicans* as efficiently as they can *E. coli* and *S. aureus* [22].

### 2.2. Biofilm and Planktonic Microbes

Biofilms are clusters of microbes (bacteria, fungi) with an extracellular matrix made up of polymeric substances, such as polysaccharides, proteins, lipids, nucleic acids, and humic substances, which attach to inert or living surfaces [23,24,25]. Extracellular polymeric substances may play the role of a protective shelter, or a diffusion barrier. Therefore, biofilms are stable enough to resist physical forces, pH changes, oxygen radicals, as well as antibiotics and phagocytosis [26,27]. Although, in some cases, the strains are comprised of different species of microbes, the biofilms produced are still stable, or often even more stable [28]. In hospitals or clinics, biofilm formation on the surfaces of medical instruments may cause nosocomial infections [29]. Similarly, such formation on the surfaces of implants lead to orthopedic implant infections [30].

Planktonic microbes are free-living microbes, which may float or swim in a fluid medium. Compared to biofilms, it is generally believed that planktonic microbes are more susceptible to antimicrobial agents such as NPs [31,32], because NPs must aggregate and interact with the extracellular polymetric substances produced in biofilms, thereby decreasing their toxicity to microbial cells [33]. Thus, the antibiotic resistance of biofilms is much greater than that of planktonic bacteria [34].

### 2.3. Biofilm Formation and Prevention

**Figure 2:**
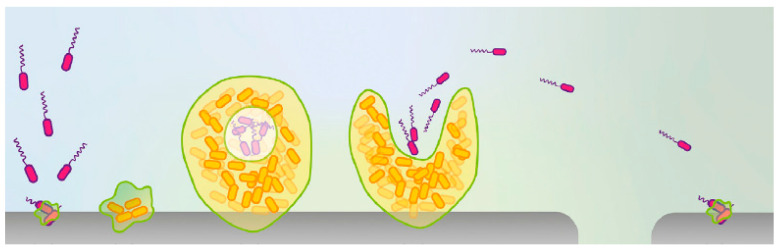
Life cycle of biofilm. Reproduced with permission from Ref [24]. Copyright 2019, Elsevier.

Nosocomial infections are a significant source of human morbidity and mortality, which affects millions of patients annually [35]. It is generally believed that planktonic bacteria attaching to the surfaces of medical devices, or public items in hospitals, may proliferate to form the initial thin biofilm. When growing to mature biofilms, planktonic bacterial cells may disperse, attacking new surfaces, and starting new life cycles (Figure 2) [24]. Ultimately, biofilm-caused contamination may spread to some key hospital areas, such as intensive care units (ICUs) [36].

**Figure 3:**
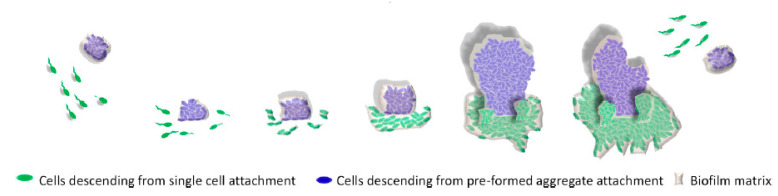
New model of biofilm formation. Reproduced with permission from Reference [37]. Copyright 2016, American Society for Microbiology.

Recently, another model was proposed to explain biofilm formation (Figure 3). This model proposes that multicellular aggregates can form biofilms more easily than single cells [37]. This model is more likely to correspond to biofilm formation in natural environments, in which the microbes form and disperse biofilms in the pattern of multicellular aggregates, instead of single cells [37].

Implants (e.g., mesh [38], dental [39], breast [40]), and other prostheses [30], also risk biofilm contamination. A mechanism of bacterial attachment on implant surfaces was proposed, involving a two-phase attachment process: physical factors, including Brownian motion, van der Waals attraction, and surface electrostatic forces, contribute to the initial phase of the interaction, followed by molecular reactions between the implant surface and the bacterial surface polymeric structure, which can result in stronger interfacial adhesion [41,42,43,44].

Patients, following surgery, may have to face the serious consequences of nosocomial and postoperative infections, and their associated high health costs [45]. Microbes, accumulated on implant surfaces, can trigger tissue inflammation, which results in osteolysis, and even bone loss [46]. Because biofilm-mediated infections on implants occur inside human bodies, they are difficult to detect and treat, so that the best method to resolve this problem is to prevent biofilm formation in the first place. Designing antimicrobial implant surfaces, such as by coating them with NPs, is currently being studied [47,48]. Implant surfaces, functionalized in this manner, were found to have not only bactericidal properties, but also resistance to bacteria adhesion [49]. There are various explanations for why nanostructured surfaces are thought to prevent biofilm formation. The sharp edges of nanostructures may destroy microbial membranes, and would also be toxic to human cells [50]. Theoretically, the hydrophilicities of the substrate and different microbial cell surfaces are related to microbial adhesion [46]. Surfaces coated with a high density of NPs can limit the adhesion of Gram-positive bacteria, because of the rigid peptidoglycan membrane, which is difficult to flex and adapt to the nanostructured surface [51]. Roughness is also believed to be related to biofilm formation: surfaces with elevated rugosities favor biofilm formation [46]. Apart from surface structure-based antimicrobial activity, NPs also have intrinsic antimicrobial mechanisms.

## 3. Antimicrobial Mechanisms

Although the precise antimicrobial mechanisms of NPs are still not known, several hypotheses have been proposed, such as the release of metal ions [12,52], antimicrobial behavior mediated by reactive oxygen species [53], direct interaction between NPs and microbes (i.e., contact killing) [54], a combined (comprehensive) mechanism [55], and immunostimulatory effects [56]. The antiviral potential of NPs is discussed in this section, as well.

### 3.1. Released Ions

**Figure 4:**
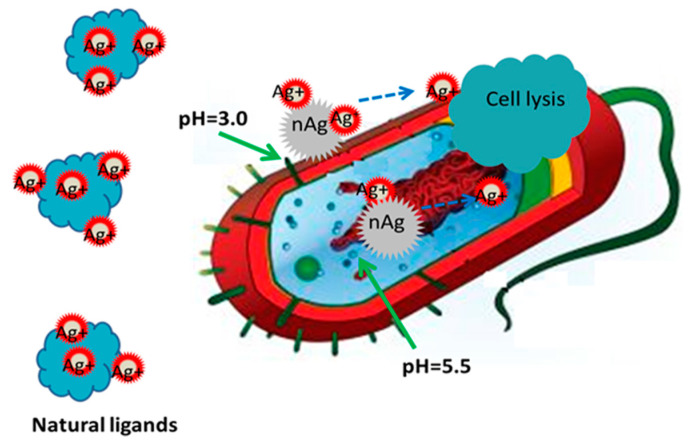
Ag NPs function as vehicles to deliver Ag ions to interact with bacterial cytoplasm and membrane, decreasing the local pH. Reproduced with permission from Reference [61]. Copyright 2012, American Chemical Society.

Many research groups [57,58,59,60] believe that the main antimicrobial mechanism is the release of ions from NPs. That is, Ag NPs only function as vehicles to transport and deliver Ag ions for interaction with bacteria, in which the Ag ions exerted the main antimicrobial effect (Figure 4) [61]. In order to clarify the antimicrobial mechanism, the antimicrobial property of Ag nitrate solution was evaluated against *E. coli* [62], which found that Ag ions interact with membrane proteins to change the membrane permeability. The mechanism of protein deactivation is probably dependent on the reaction of Ag ions with cysteine residues [63]. After the released ions enter bacterial cells, DNA and RNA, and their transcriptional responses, are affected [57]. In other research, in order to eliminate the influence of the NPs themselves, NPs were confined in a matrix, which only permitted ion generation and release [64]; the results showed good antibacterial properties, which demonstrated the key role of ions in antimicrobial activity.

It was also shown that antimicrobial efficacy is based on the surface charge of Ag NPs, in which positively charged Ag NPs exhibit higher antimicrobial effectiveness than those negatively charged [65]. Generally, the surface charge can be altered through conjugating different capping agents. The cell walls of both Gram-positive [66] and Gram-negative [67] bacteria are negatively charged. Therefore, the greater the positive NP surface charge, the lower the electrostatic barrier. As a result, positively charged Ag NPs interact more readily with bacteria and exhibit greater antibacterial properties [68].

### 3.2. Reactive Oxygen Species

Reactive oxygen species (ROS) are short-lived, highly reactive molecules containing oxygen. Typically, they consist of unstable oxygen free radicals, including hydroxyl radical (•OH), peroxide (O_2_•^−2^) and superoxide (O_2_•^−^) anions, and non-radicals, such as hydrogen peroxide (H_2_O_2_) and hydroxyl ions (OH^−^) [69,70]. Normally, ROS are generated and consumed by cells under dynamic balance. If the generation of ROS surpasses the antioxidant capacity of microbial cells, oxidative stress may be induced [71]. Such oxidative stress is liable to damage intracellular biomacromolecules, such as proteins, lipids, RNA and DNA [72,73,74,75,76].

Different ROS exhibit different antimicrobial capacities. The commonly discussed ROS in antimicrobial activity are OH^−^, H_2_O_2_, and O_2_•^−^. Some negative ROS, such as OH^−^, are prone to interact with positively charged microbial cell membranes, although H_2_O_2_ is more efficient in penetrating cell membranes [77,78]. Interestingly, one report indicates that ROS can maintain cell membranes intact and simultaneously destroy intracellular biomolecules [79].

**Figure 5:**
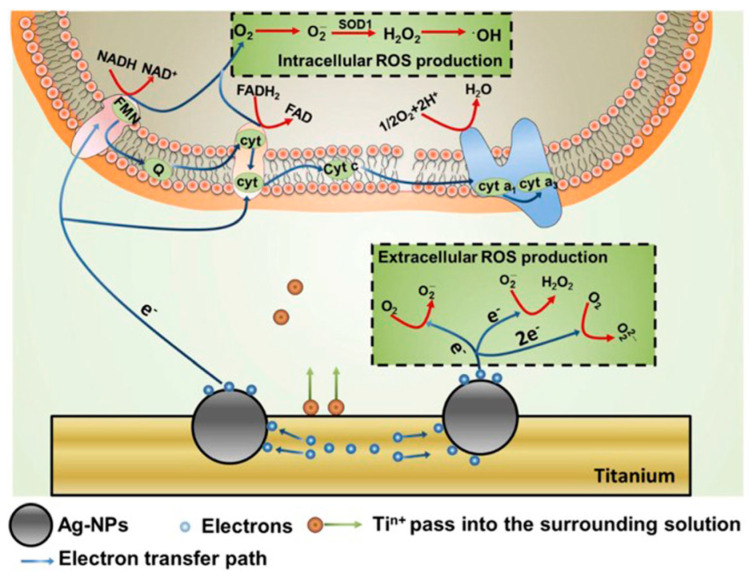
Production of ROS outside and inside bacterial cells. Reproduced with permission from Reference [80]. Copyright 2017, Elsevier.

Ions released from NPs under humid circumstances can induce ROS generation. Electrons released from Ag NPs have been found to lead to bursts of ROS in both extracellular and intracellular environments (Figure 5) [80]. The oxidative stress induced by excess ROS can destroy biomolecules; once a ROS scavenger, such as acetylcysteine, is added, the antimicrobial activity of Ag NPs is noticeably restrained, confirming that bacteria can be killed by NP-induced excess ROS production.

### 3.3. Contact Killing

**Figure 6:**
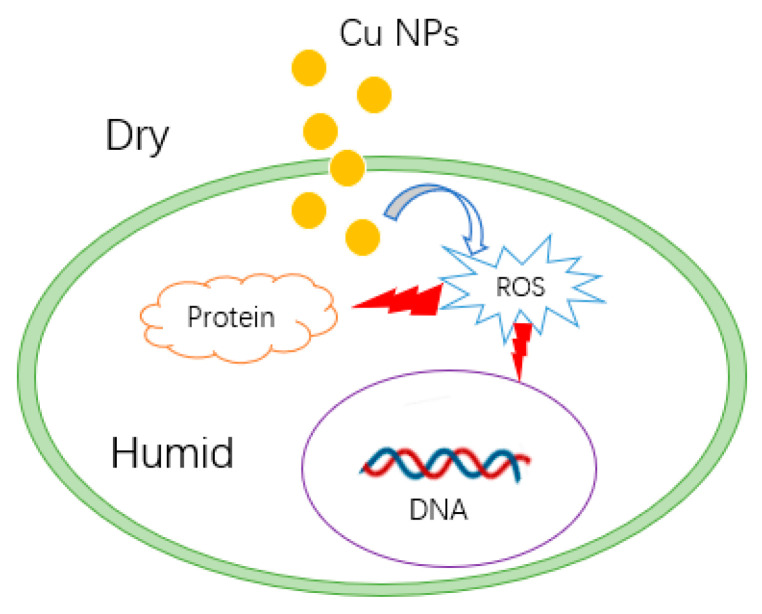
Possible contact killing mechanism of Cu nanoparticles (NPs).

Some research has demonstrated that NPs possess antimicrobial properties under dry conditions [81,82], indicating that direct contact appears to be a potential antimicrobial mechanism. In a dry environment, no electrochemical reactions occur on the surface of NPs, so ions and electrons are not released to interact with biomolecules, or induce ROS bursts. It is posited that Cu NPs interact with membrane proteins [83], and penetrate into bacterial cells [84], inducing an explosion of ROS in the intracellular environment (Figure 6) [85]. Experiments have shown that the production of ions cannot increase the antibacterial effect of Cu NPs [81].

In some studies, Ag NP were found to show good antimicrobial properties under circumstances where no Ag ions could be detected [86]. Additionally, control experiments were carried out to compare the antimicrobial efficiencies of Ag NPs and Ag ions, which indicate that NPs were more effective against *E. coli* than ions [87]. It was reported that the concentration of released ions from different sizes of Ag NPs was essentially identical, while their antibacterial activities were different, implying that contact killing was the dominate antibacterial factor [88]. Another example, based on a comparison between immobilized and colloidal Ag NPs [89], also implied that contact killing was the predominant antimicrobial mechanism. As well, the formation of porous structures and holes on the *E. coli* cell surface, when attaching to Ag NPs, is evidence of contact killing [90]. However, most studies were not conducted under absolutely dry conditions, which would have eliminated the possible effect of ions and ROS.

### 3.4. Combined Antimicrobial Mechanism

NP antimicrobial activity does not appear to be dependent on any one individual hypothesis. Rather, the previously cited mechanisms (NPs, released ions, and ROS) should all be considered, with several possibly operating synergistically in the antimicrobial process.

**Figure 7:**
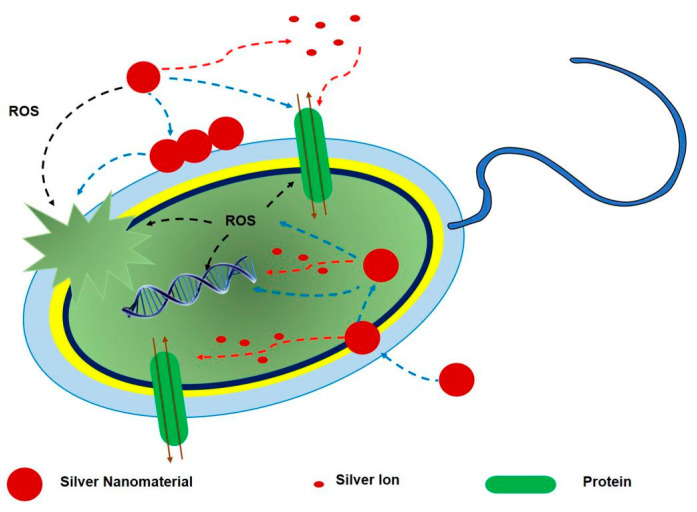
The comprehensive mechanism of antibacterial activity of Ag NPs. Reproduced with permission from Reference [55]. Copyright 2018, John Wiley and Sons.

In terms of this hypothesis, all may simultaneously exercise their own separate roles [55,91,92]. As an example, Ag NPs may accumulate on the bacterial cell walls and membranes, and regulate membrane proteins [93], changing the membrane permeability to permit both Ag NP and ion transport into bacteria cells. Ag NPs that have penetrated into cells, continue to release ions, which can attack proteins and DNA. Intracellular ROS are produced by Ag ions, which may also affect proteins and DNA (Figure 7) [55].

### 3.5. Immunostimulatory Effects

In addition to direct killing, NPs can also modulate immune responses, and enhance innate antimicrobial immune defenses [56]. Reactive nitrogen species (RNS), like ROS, are important in the antimicrobial process [94]. Ag and Cu NPs can cause an increase in the concentration of nitric oxide, one kind of RNS, resulting in a synergistic host immune defense against microbes [95,96]. Nitric oxide can also oxidize Cu, present in protective proteins in microbes, to free Cu ions, which boosts toxicity to microbial cells [56].

In addition to RNS, antimicrobial peptides are abundant natural antibiotics, produced by humans, which play a significant antibiotic role in the immune system [97,98]. Both Ag and Cu NPs exhibit synergistic antimicrobial effects with polymyxin B, one type of antimicrobial peptide [56,99].

Adjuvants are often used in vaccine production to improve the immune response [100]. Ag NPs, used as vaccine adjuvants, can dramatically induce the increase of the antigen-specific IgG1/IgG2a ratio, as well as antigen-specific IgE. Local leukocytes, particularly macrophages, are also activated by Ag NPs [101].

Although the immunostimulatory effect of NPs has been proposed as a possible antimicrobial mechanism, the relevant reports are still scarce.

### 3.6. Antiviral Mechanism

In the context of the COVID-19 pandemic and its ongoing vaccine development, research on effective antiviral agents is urgent. It has been reported that the survival time of the coronavirus on different materials, such as metal, paper, plastic, and glass, varies from a few hours to days [102]. Because of their ability to interact with proteins, DNA and RNA, metal NPs have the potential to destroy viruses.

**Figure 8:**
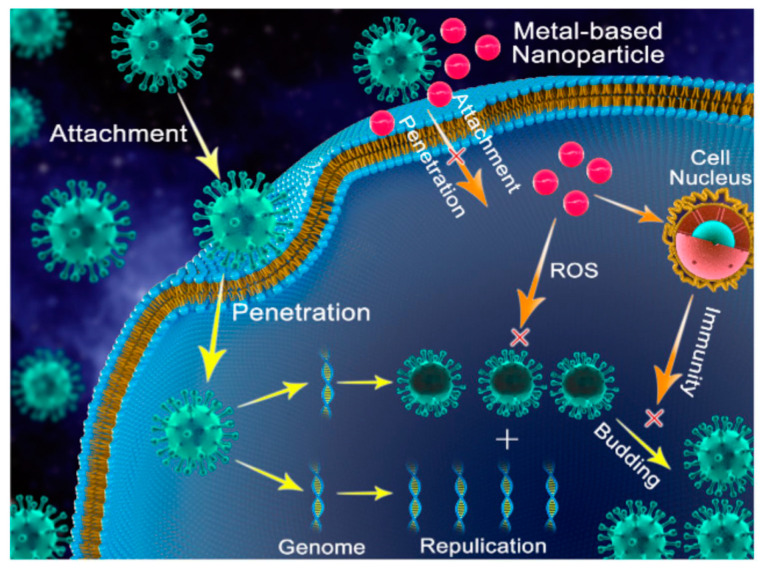
Possible antiviral mechanism of metal NPs. Reproduced with permission from Reference [103]. Copyright 2020, Springer Nature.

One possible NP antiviral mechanism was proposed as occurring in three stages (Figure 8): (1) interaction with the viral protein shell, to restrain its attachment to human cells; (2) production of ions and ROS, which destroy the viral protein shell, and DNA or RNA; (3) NP penetration into the cell, followed by interaction with enzymes, to prevent viral replication and subsequent spread [103,104].

Ag NPs have been shown to have a high antiviral capability toward the African swine fever virus, through interacting with its protein shell, and thereby preventing viral penetrating into the animal’s cell [105]. One study, involving Ag_2_S NPs, indicated that the antiviral capacity predominantly influences the stages of viral RNA replication and budding [106]. Both naked Ag NPs and those coated with polysaccharide, poly N-vinyl-2-pyrrolidone, and mercaptoethane sulfonate, exhibit activity toward HIV, TCRV, RSV, HBV, MPV, and HSV viruses [107]. Due to their broad-spectrum antiviral properties, Ag NPs have the potential to be used as antiviral drug.

Cu NPs also display excellent antiviral behavior. Cu was found to inactivate norovirus through inhibiting its RNA replication while its protein shell remained intact, which suggests that ions penetrated into the virus to act against the RNA [108]. CuO NPs, with an average size of 40 nm, exhibited effective inhibition of *Herpes simplex* virus type 1 (HSV-1), although the antiviral effectiveness was not as good as that of the conventional antiviral medicine, acyclovir [109].

## 4. Ag and Cu NPs

The difference in antimicrobial ability between Ag and Cu NPs was initially thought to depend on the different amounts of ions released [110]. The activity of Cu was found to be greater than that of Ag, and, at the same NP concentration, ions released from Cu NPs were found to be at a higher concentration [111]. However, the antimicrobial ability of Ag NPs was found to be greater than that of Cu NPs, indicating that Ag ions are more efficient in antimicrobial activity than Cu ions [111,112]. Ag NPs also show broader antimicrobial effectiveness to various strains of *E. coli* and *S. aureus*, as well as to fungi [113], which may be due to their stronger interaction with polysaccharides and proteins on cell walls [114]. The existence of an oxide layer on Cu NPs was proposed to be the reason that the antimicrobial capacity of Cu NPs is less than that of Ag NPs [115,116].

### 4.1. Influence of Size and Shape

Size has a considerable influence on antimicrobial properties. For a given mass, the smaller the NP size, the higher the surface:volume ratio, which increases the antimicrobial capacity, as ions can be more rapidly released [63,79,117]. Ag NPs synthesized from green and black tea leaf extracts have shown superior antimicrobial properties than Cu NPs produced by the same method, because the size of the Ag NPs produced is smaller [118]. However, others have suggested that size does not have much of an influence on the antimicrobial properties, rather that surface charge is the most significant influence factor [65]. Another study showed that larger sized Ag nanoclusters can release higher concentrations of ions, although the antimicrobial effect was not influenced to a great extent [119]. However, size may not be the most significant antimicrobial factor, as another study revealed that larger Ag NPs are more effective than smaller ones [65].

Nanocrystal shapes are produced by controlling growth speeds along different crystallographic directions [120]. Antimicrobial properties are also impacted by different NP shapes. Truncated triangular and spherical Ag NPs are more effective than Ag nanorods [121]. Another study reached a similar conclusion: hexagonal Ag NPs, similar to truncated triangular NPs, show better antimicrobial effects than spherical and triangular shapes [122]. These results may indicate that antimicrobial effects are not related to size, because the weakest triangular NPs have the largest surface areas at a given volume. If the surface area determined antimicrobial properties, smaller sized NPs, with higher surface areas, would have stronger antimicrobial activities. However, this is not what was found. One study indicated that the (1, 1, 1) facet might be able to enhance antimicrobial property because it can generate singlet oxygen (one type of ROS), under photo-irradiation, while other facets do not have this function [120,123]. Anisotropic Ag NPs exhibit higher antimicrobial effects than spherical ones, which is attributed to a greater number of crystal (1, 1, 1) facets.

In addition to size and shape, the existence of corners, edges, defects and deformations on the microstructure may also influence the antimicrobial effect [110].

### 4.2. Influence of Surface Treatment

Chemical agents or surface treatments may enhance the antimicrobial abilities of NPs. In one study, Cu-loaded silica nanomaterial showed improved antimicrobial efficacy over bare Cu NPs, while Ag-loaded silica did not show better antimicrobial ability, when compared with plain Ag NPs [124]. Surfactants, including SDS, Tween 80, and PVP, can stabilize NPs against aggregation, which can enhance their antimicrobial properties [125]. Cu NPs, coated with starch macromolecules, showed more efficient antimicrobial efficacy than Ag NPs, the starch coating being capable of reducing oxidized Cu [126]; other coatings did not influence their antimicrobial properties. Both Ag and Cu NPs were grafted onto the surfaces of carbon nanotubes; the Ag-grafted carbon nanotubes were found to have greater antimicrobial properties than the Cu-grafted ones, while the pure carbon nanotubes had the poorest antimicrobial performance [127]. In the case of argon plasma surface treatment, the antimicrobial effect of polymer surfaces coated with Cu NPs was greater than those coated with Ag NPs, perhaps because the surface roughness of the polymers coated with Cu NPs was greater than that of the surfaces coated with Ag NPs, resulting in greater exposure to microbes [60].

To overcome microbial resistance to either NPs or conventional antibiotics, the functionalization of NPs with antibiotics appears to be promising. The synergistic antimicrobial effect of the combination of Ag NPs and antibiotics was found to be much greater than Ag NPs or antibiotics, alone [128,129]. Similar to Ag, Cu NPs, combined with various antibiotics, particularly ampicillin, had increased antimicrobial properties [130]. The enhancement of microbial susceptibility to the combination of NPs and antibiotics may result from the higher permeability of microbial cell walls, modulated by NPs, which can facilitate the entry of antibiotics into cells; another reason may be that the enzymes, which play key roles in the antibiotic resistance, are inactivated by NPs [131].

### 4.3. Oxide NPs

In general, both Ag oxide and Cu oxide NPs are considered to belong to the set of Ag and Cu NPs, because Ag and Cu NPs oxidize when exposed to atmosphere. Theoretically, Cu NPs are easier to oxidize than Ag [111].

Despite the low number of articles on antimicrobial Ag oxide (i.e., Ag_2_O and Ag^I^Ag^III^O_2_) NPs, they appear to show antimicrobial properties [132,133,134]. It is believed that AgO is the most active against microbes [8]. In contrast to Ag oxides NPs, there is a large number of reports on Cu oxide NPs, including both Cu_2_O and CuO. For Cu NPs deposited onto the surface of TiO_2_, they were shown to be covered with a thin mixed CuO, Cu_2_O layer [29]. The mechanism of contact killing, described earlier, deals mostly with these NPs. It is supposed that the antimicrobial effect of CuO NPs depends on the production of •O_2_^−^ ROS [135]. However, Cu_2_O is considered to be the more effective agent, forming a copper(I)-peptide complex; the inactivation of proteins caused by Cu_2_O NPs cannot be detected when using CuO NPs [136]. Although CuO NPs can generate ROS while Cu_2_O cannot, the antimicrobial efficacy of Cu_2_O is, nonetheless, greater [136]. This conclusion may be evidence for the contact kill mechanism, as ROS do not work as efficiently as copper(I)-induced protein inactivation. Although CuO NPs also have antimicrobial properties when compared with Ag and Cu NPs, higher concentrations are required to attain the same antimicrobial efficacy [137,138]. In general, preventing oxidation is an efficient way to enhance the antimicrobial properties of Cu NPs.

### 4.4. Other Derivative NPs

Apart from oxide NPs, the main Ag-derived NPs are AgX (X = Cl, Br, I). Many studies posit that the antimicrobial mechanism of AgX NPs is related to their photocatalytic activities [139,140,141]: it was reported that AgBr NPs can form electron/hole pairs when irradiated with visible light at 400 nm, which may induce ROS production [142]. Another study posited that the antimicrobial behavior of AgCl and AgI NPs is based on the release of Ag ions, not noticeably different from antimicrobial Ag NPs [143]. However, because of their photocatalytic properties, AgX NPs are unstable under visible light irradiation, which results in the decline of their antimicrobial behavior over time [144]. Antimicrobial activities have also been reported for other Ag-related NPs, such as Ag_2_S [145] and Ag_2_Se [146].

In addition to Cu oxide, CuS [147] is the main Cu-based NP; it, too, displays antimicrobial behavior. It was found that CuS NPs, at specific wavelengths (980 nm [148] and 808 nm [149]), have strong photothermal effects, which can be used to kill bacteria. However, another study demonstrated that CuS NPs possess higher antimicrobial potency than Cu ions in the absence of light irradiation [150]. CuS-damaged cell walls were detected, because bacteria do not evolve to be resistant to membrane-disrupting antibiotics [151].

### 4.5. Bacterial Susceptibility

Different bacteria show different susceptibilities to Ag and Cu NPs. It was found that these NPs have equal antibacterial behavior toward Gram-positive bacteria [152]. A similar conclusion was obtained in another study, where *B. subtilis,* a Gram-positive bacterium, had approximately equal sensitivity to both Ag and Cu NPs, although Cu NPs demonstrated a higher antimicrobial efficacy to the Gram-negative *E. coli* [153]. However, the opposite results were obtained in another report, which revealed that Ag NPs were more effective toward *E. coli* and *S. aureus*, while Cu NPs showed better antimicrobial action toward *B. subtilis* [115]. Similar results concluded that Cu NPs are more effective to the Gram-positive *B. subtilis*, while Ag NPs have superior antimicrobial activity against the Gram-negative *E. coli* [154]; thus, one cannot formulate an exact conclusion based on a comparison between Ag and Cu NPs toward different bacteria.

Some types of bacteria are resistant to Ag and/or Cu [155,156,157]. Cu-resistant bacteria exist extensively in Cu-contaminated soil [158,159]. One kind of Gram-positive bacterium, *Ent. faecium*, showed strong resistance to both Ag and Cu ions, probably because bacterial cellular Cu homeostasis resulted in Ag efflux [160]. However, the Gram-negative Cu-resistant bacterium, *E. coli*, is sensitive to Ag, the mechanism of which may be different from that of *Ent. faecium* [160]. Another study proposed nine genes in three transcription units, which could cause *Salmonella* resistance to Ag compounds [161]. A similar viewpoint proposed that Ag-resistant *E. coli* had a reduced outer membrane permeability, which was presumably determined by a chromosomal gene [162].

### 4.6. Cytotoxicity to Human Cells

NPs can release ions, induce intracellular ROS generation, or directly interact with cells, all of which are likely to cause cytotoxicity to human cells. In addition, the accumulation of toxic NPs in the environment would increase the possible environmental risk. Thus, the potential cytotoxicity of NPs to human must also be considered.

It is believed that Ag and Cu ions released into the environment are toxic to the human liver, kidney, eye and skin [163,164]. Generally, the cytotoxicity of NPs to human cells is also related to NP size, since NPs with large surface to volume ratios release more ions than bulk metals [165]. Apart from ions, an increasing amount of intracellular ROS induced by NPs may trigger cells death [166]. The ROS bursts and pro-inflammatory pathways induced by Ag NPs may lead to DNA damage, protein misfolding, and lipid peroxidation, all of which are possibly unrepairable, and potentially carcinogenic [167]. The effect of Ag NPs on the immune system should be further studied, since one report suggested that Ag NPs exerted cytotoxicity on macrophages, and resulted in an inflammatory response and cellular apoptosis [168]. Unlike Ag, Cu is a necessary element for the human body, where it must be retained in homeostasis. If the Cu content increases to break the equilibrium, it becomes toxic [169].

However, a histological study indicated that Ag and Cu NP coatings on catheters do not irritate the human skin [170]. It was also found that Ag-functionalized polyurethane is toxic to bacteria, but not to cells, when the Ag NP content is optimized to 0.5 wt %, at which cells vitality is near 100% [171]. This is attributed to the serum protein albumin in the cell culture medium, which reduces the biological interaction of the NPs with the cells [171]. The restraint to NPs by the immune system can result in a greater resistance of human cells, compared to microbes, to Ag NPs, which suggests that Ag NPs, at antibacterial concentrations, may be nontoxic to human cells [172]. It was also reported that newly produced Ag NPs are less toxic to human cells than older ones [173]. Immobilizing NPs can reduce their toxicities, compared to free NPs [174]. Titanium substrates, coated with chitosan, hydroxyapatite, and Ag NPs, revealed strong antimicrobial capacities and weak cytotoxicity to humans, since chitosan immobilizes Ag NPs firmly, thereby reducing cytotoxicity to human cells [175].

## 5. Coatings on Substrates

Besides being used as aqueous disinfectants [176,177], the main application of NPs is as coatings on substrates, such as textiles (synthetic polymers and cotton) and implants (polymers, ceramics and metals). Other products, including food packaging materials [178,179], and water [180,181] and air [182,183] filters, can also be decorated with antimicrobial NPs.

### 5.1. Coatings on Textiles

The COVID-19 pandemic has motivated work on antimicrobial NP-functionalized textiles, especially for use as face masks and protective clothing. Although the antiviral properties of Ag and Cu NPs are not clear, the NP decoration can improve the protective function of these masks [184]. N95 masks, impregnated with Cu oxide NPs, were reported to filter 99.85% of aerosolized viruses, such as human influenza A virus (H1N1) and avian influenza virus (H9N2), without reducing physical filtration performance [185]. In addition to viruses, textiles are common substrates for bacterial growth, under proper temperature and humidity condition [186]. Those impregnated with Ag and Cu NPs had dramatically enhanced antibacterial properties when compared to unimpregnated PET textile [187]. Similarly, studies on cotton textiles impregnated with Ag NPs, and those impregnated with a mixture of Ag and Cu NPs, both exhibited excellent antibacterial and antifungal properties [188]. It was initially hypothesized that the antimicrobial properties are dependent on both the textile and the NP [189], although it was subsequently demonstrated that the antimicrobial efficacy has nothing to do with the textile type, but depended only on the NPs [190].

Many methods of coating onto textiles have been reported. For instance, NP dispersions can be coated onto cotton fibers by means of pad dyeing [132], immersing [191], or ironing [190], all of which are traditional coating methods in the textile industry. Chemical reduction [192] is another commonly used treatment method: Ag and Cu NPs can be deposited onto PDA/PET fabrics by the chemical reduction of aqueous Ag and Cu salt solutions [187]. However, the problem for NPs loaded onto textiles is that they are poorly bonded, and tend to fall off during washing, leading to the loss of antimicrobial properties.

### 5.2. Coatings on Implants

Compared to the research on textiles, NP-functionalized implants (polymers, ceramics and metals) intended for the human body, must meet stricter standards of biocompatibility and biotoxicity.

Polyurethane is one of the most frequently used polymers in the biomedical field. It has been reported that polyurethane catheters can be Ag- and Cu-functionalized by sputtering, boosting its antimicrobial properties [116]. In addition to polyurethane, polyethylene is usually used in joint arthroplasty, owing to its remarkable mechanical properties, though it is easily attacked by microbes. In a study on polyethylene surface modifications, polyethylene coated with Ag NPs possessed greater antimicrobial properties than that coated with Cu NPs [193], which corresponds to the antimicrobial comparisons between Ag and Cu NPs discussed earlier. Other polymers, such as polytetrafluoroethylene (PTFE), used as an implant due to its good chemical resistance and thermal stability, were also coated with Ag and Cu NPs to improve their antimicrobial properties [194].

Bio-ceramics include bioinert, bioactive, and biodegradable ceramic materials. Bioinert ceramics are nontoxic, and do not interact with human tissue. Bioactive ceramics, such as bio-glass and hydroxyapatite, generate new bonds to human tissue. Biodegradable ceramics, such as calcium phosphates, are resorbed by the human body [195]. Here, we discuss bioinert ceramics, such as TaN, TiO_2_, etc. Tantalum nitride (TaN), modified with both Ag and Cu NPs, displayed greater antimicrobial activity than either TaN-Ag or TaN-Cu, because of the synergistic effect of Ag and Cu NPs [196]. Another study reported that Cu NP-functionalized TiO_2_ substrates do not exhibit antimicrobial activity, whereas both Ag NPs and AgCu alloy NPs show excellent antimicrobial properties, attributed to a substantially lower release of Cu ions [197].

Titanium (Ti) metal is widely used for orthopedic and dental implants. It was hypothesized that Ti substrates embedded with Ag NPs have enhanced electron transfer between Ag and Ti, which generates ROS to kill microbes [80]. It was noticed that, in some cases, Ti substrates were treated to form TiO_2_ [197], or coated with hydroxyapatite [175], before further modification with NPs, in order to form porous surfaces that more firmly immobilized NPs.

Stainless steel is another metal used for medical applications, because of its distinct mechanical and corrosion-free features. Functionalized with Ag NPs, stainless steel showed stable antimicrobial activity, even over seven cycles of bacterial application [198]. Stainless steel, functionalized in this manner, was found to have not only bactericidal properties, but also resistance to bacteria adhesion [49].

Magnesium and its alloys are also potential implant materials. Magnesium may be intrinsically weakly toxic to microbes, since its corrosion creates an alkaline product [199]. In order to improve the antimicrobial property of magnesium, Cu NPs were coated onto its surface [200].

### 5.3. Surface Coating Methods on Implants

Various methods have been used to functionalize implants, including physical, chemical, and plasma depositions.

Magnetron sputtering is one of the widely used physical deposition methods, which results in uniform thickness, as well as strong bonding to substrates [201]. This method is generally used to produce thickness-controlled NP coatings [202]. An interesting study indicated that longer sputtering times are associated with enhanced cytotoxicity [203]. Hence, the determination of an optimal sputtering time is indispensable.

Chemical reduction on the substrate surface is the simplest and most widely used method. Ag NPs, depositing on the substrates, can be formed through the chemical reduction of metal salt solutions, which is similar to the functionalization of fabrics, described above, although the bond strength between NPs and these substrates is not high [201]. Reduction can be triggered by chemical agents [204,205], UV irradiation [206], etc. The antimicrobial properties of Ag NPs are enhanced with increased Ag salt solution concentrations [207]. It was found that the sequence of adding Ag salt solution and reducing agent influences the uniformity of surface modification [119], and may play a role here, too.

Plasma immersion ion implantation (PIII) is the most economical and effective, and involves positive ions vertically incorporating into a negatively charged surface under an electric field [208]. Thus, NPs can be impregnated into the near-surface of implants, including polymers, ceramics and metals [201]. The average size of Ag NPs incorporated into substrates increased with increased PIII time [198,209], because of aggregation. However, PIII was found to reduce Ag NP cytotoxicity by constraining NP mobility on titanium substrates [210].

Plasma electrolytic oxidation (PEO), also called micro-arc oxidation (MAO), is another commonly used plasma deposition method. PEO developed from anodization, forms ceramic-like coatings on substrates, which can tightly immobilize NPs onto surfaces [211]. PEO is often used to generate a porous titanium dioxide thin layer on titanium or titanium alloys, for further surface modification with NPs [212,213]. Magnesium alloys, implanted with Cu NPs through PEO, inhibited bacterial proliferation more efficiently than the alloy treated by PEO without Cu NPs [200].

Table 1 summarizes methods of implant surface modification with Ag NPs, Cu NPs, Ag–Cu NP mixtures, and AgCu nanoalloys. As can be seen, PIII is generally used to functionalize NPs on metals and polymers, while magnetron sputtering is usually employed for the surface modification of ceramics and polymers. Compared to PIII and magnetron sputtering, PEO can generate much thicker porous oxide layers, incorporated with NPs, on metal substrate surfaces. Since polymeric materials may contain various active functional groups, chemical reduction is not suggested for their modification. Apart from these, other surface functionalization methods, such as heating organic solvents [194], anodization [206], adding linkers on substrates [214], and electrodeposition [215], have been reported.

## 6. Mixed Ag–Cu NPs and AgCu Nanoalloys

Although both Ag NPs and Cu NPs exert significant antimicrobial properties, mixtures of Ag and Cu NPs exhibit greater antimicrobial properties than either individual Ag or Cu NPs [196], which indicates the existence of synergistic antimicrobial behavior [197,223]. The mechanism of this synergy has been studied through a consideration of the nanocrystalline microstructures formed. In this section, the synthesis of AgCu nanoalloys, microstructure, and physicochemical characterization, as well as the synergistic antimicrobial mechanism, are discussed.

### 6.1. Synthesis of AgCu Nanoalloys

The most conventional AgCu nanoalloy synthesis method is through the chemical reduction of a solution containing both Ag and Cu salts. Nanoalloys were synthesized from a solution of AgNO_3_ and CuSO_4_, using natural reducing agents, such as fruit peel extract [224], or Azadirachta indica leaf extract [225], as well as synthetic reducing agents, such as polyvinylpyrrolidone [9], polyol [226,227], ascorbic acid [228], dextrose [229], sodium borohydride [190], or tartaric acid [152]. Another study used oleyl amine as both reducing agent and surfactant, to synthesize nanoalloys from a solution of Ag and Cu(I) complexes, which gave a randomly distributed AgCu solid solution [230]. The chemical reduction method is also used to synthesize AgCu nanoalloys with the microstructure of either Ag_core_Cu_shell_ or Cu_core_Ag_shell_, depending on the sequence of reduction reactions [231].

Dealloying is another widely used synthesis method. It was found that a AgCu core-shell microstructure could be generated by dealloying a Zr-Cu-Ag-Al-O crystalline composite: Zr, Al and their oxides were dissolved, leaving Ag on the Cu surface [232]. Besides core-shell microstructures, single-phase supersaturated AgCu nanoalloys were synthesized through the dealloying of Ma-(Ag, Cu)-Y metallic glass precursors in H_2_SO_4_ [233,234].

Laser ablation is also a commonly used synthesis method. Nanosecond laser pulses, at specific wavelengths, were used to generate NPs [194]. It was reported that AgCu nanoalloys could be synthesized by irradiating an unfocused laser (800 nm) on a Ag and Cu colloidal solution for a period of time, while stirring [235]. Another study used a 1064 nm laser beam to irradiate pure Ag and Cu targets in an aqueous medium, adding different concentrations of capping agent to synthesize AgCu nanoalloys having different sizes [236]. AgCu nanoalloys having different compositions have also been synthesized by laser ablation [237].

The use of galvanic displacement reactions is an effective method to synthesize AgCu nanoalloys, especially for core-shell microstructures [238]. Galvanic displacement permits Ag precursors to chemically decompose, transferring electrons from Ag ions to Cu^0^, replacing crystalline Cu atoms [239], ultimately forming Ag-doped AgCu nanoalloys. Another example, using a displacement reaction to synthesize AgCu nanoalloys, was based on the metallic activity difference between Ag and Cu, the first step of which is Cu NP generation from a Cu precursor, and the second step is Ag atom displacement of Cu atoms on the surface of Cu NPs through electrons transfer [240]. CuO microparticles, decorated with Ag NPs via galvanic displacement reactions, were also reported [241].

In addition to these methods, other synthesis approaches have been described, such as the sol-gel reaction [242], carbothermal shock [243], inert gas condensation [244], the electric explosion of twisted Ag and Cu wires [245], a core-shell structure produced by sono- and electrodeposition [246,247], and magnetron sputtering [248].

### 6.2. Microstructures of Mixed Ag–Cu NPs and AgCu Nanoalloys

**Figure 9:**
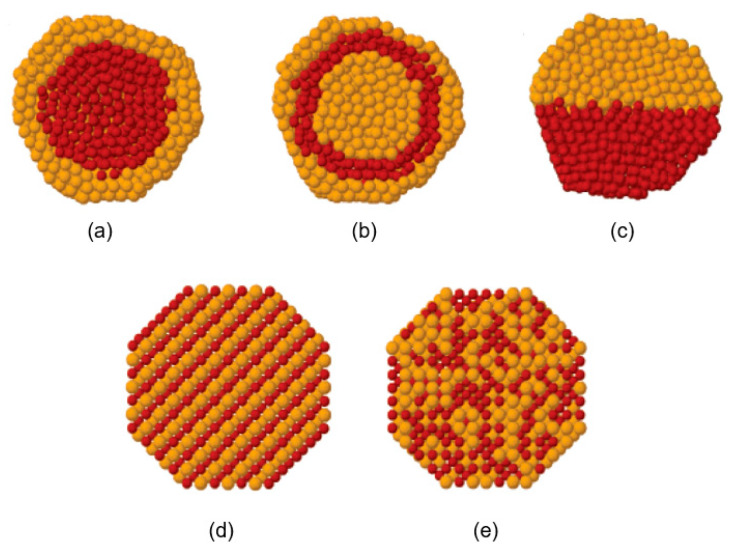
Microstructures of bimetallic NPs. (**a**) core-shell structure; (**b**) multi-shell structure; (**c**) biphase structure; (**d**) intermetallic structure; (**e**) nanoalloy. Reproduced with permission from Reference [249]. Copyright 2008, American Chemical Society.

Mixtures of bimetallic nanoparticles may form different microstructures (Figure 9) [249,250]. Segregated microstructures, with two or more separated NP clusters sharing a limited interface, include core-shell (Figure 9a), multi-shell (Figure 9b), and biphasic (Figure 9c) microstructures. Core-shell microstructures commonly exist in bimetallic NPs. In addition to segregated microstructures, mixed microstructure may be divided into an intermetallic structure (Figure 9d) with an ordered bimetallic atoms alignment, and a nanoalloy structure (Figure 9e) with randomly distributed bimetallic atoms [250].

**Figure 10:**
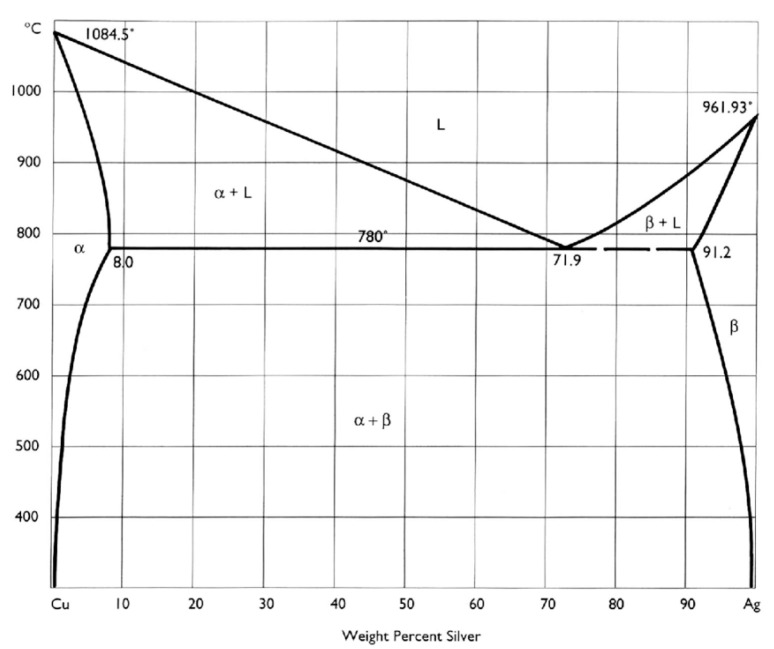
Ag–Cu phase diagram. Reproduced with permission from Reference [251]. Copyright 1991, J. Paul Getty Trust.

**Figure 11:**
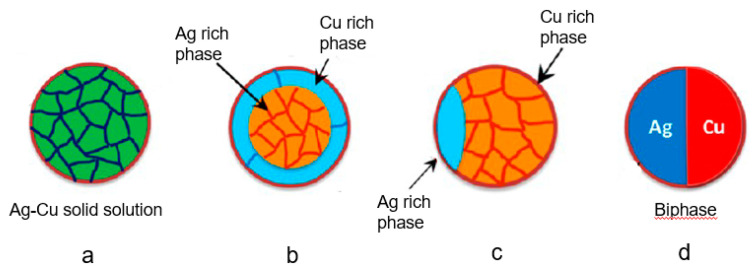
(**a**) Single-phase AgCu solid solution; (**b**) core-shell structure of AgCu nanoalloys; (**c**) structure of Ag and Cu in both phases; (**d**) biphasic. Reproduced with permission from Reference [254]. Copyright 2016, American Chemical Society.

**Figure 12:**
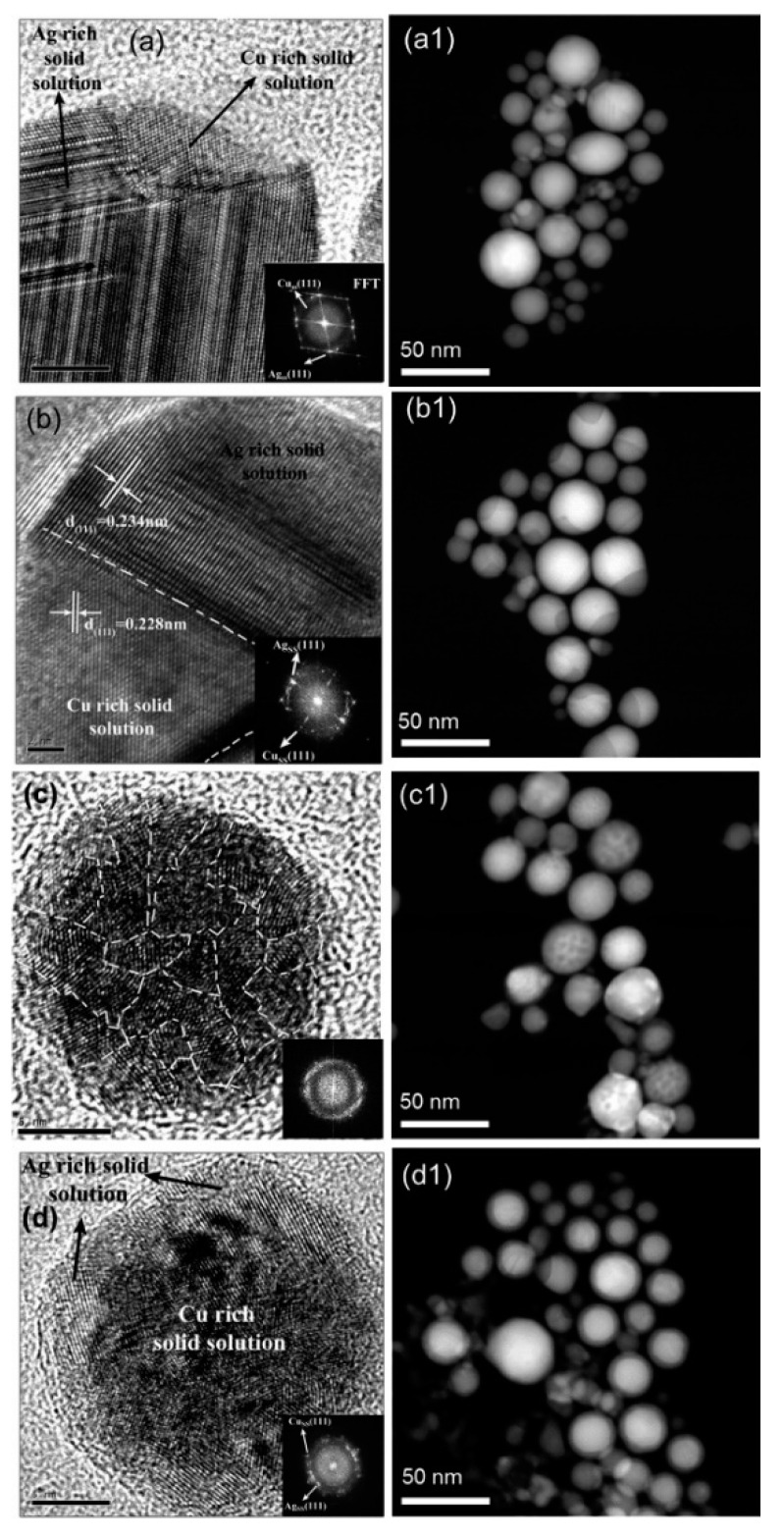
HRTEM photomicrographs of AgCu nanoalloys, showing four different microstructures: (**a**,**b**) separated phases, (**c**) nanograins and (**d**) core-shell with, respectively, four different Cu concentrations (20%, 40%, 60%, 80%). (**a1**–**d1**) images of four respective target compositions at low magnification. Reproduced with permission from Reference [237]. Copyright 2014, American Chemical Society.

As the Ag–Cu phase diagram indicates (Figure 10) [251], the metals are mutually immiscible at low temperature. Thus, mixed Ag and Cu bimetallic NPs generally form distinct phases [227,252], rather than homogeneously dispersed microstructures, as does the AuCu nanoalloy [253]. It has been reported that the Ag–Cu mixture tends to form core-shell structures, in which Ag is the shell and Cu the core. This is because Ag, with a lower surface energy (1.25 J/m^2^) segregates on the surface of Cu NPs, with their greater surface energy (1.79 J/m^2^) [190]. In an approximate sense, a mixture of Ag and Cu bimetallic NPs, with a nanograin microstructure, can be considered a single-phase nanoalloy (Figure 11a), whereas, in other microstructures, such as in Figure 11b,c, the Ag and Cu atoms do not distribute uniformly. It was found that homogenous AgCu nanoalloys (Figure 11a) gradually separate phases (Figure 11b–d), as the temperature is increased [254]. In addition to the influence of temperature, the morphologies of the Cu-rich α phase and the Ag-rich β phase can change with an increase of Cu content, passing from separated phases to nanograins to core-shell microstructures (Figure 12a–d), since the microsystems tend to keep the α-β interphase surface energy minimum [237,255]. In a broad sense, all the microstructures described here belong to AgCu nanoalloys, while only the simply mixed Ag–Cu system, without any combination reaction, is not so regarded.

**Figure 13:**
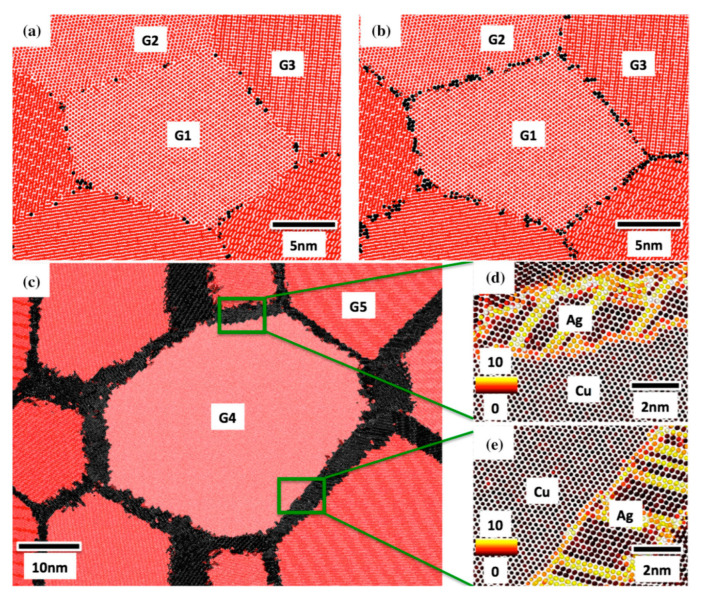
Microstructures of Ag doping Cu grain boundaries; (**a**–**c**) samples are based on the increases in Ag concentration; (**d**,**e**) are the Cu/Ag interfaces. For (**a**–**c**), red denotes Cu and black denotes Ag; for (**d**,**e**), the perfect FCC structure is marked 0, the light color, while defects are marked 10, the black color. Reproduced with permission from Reference [256]. Copyright 2017, Springer Nature.

**Figure 14:**
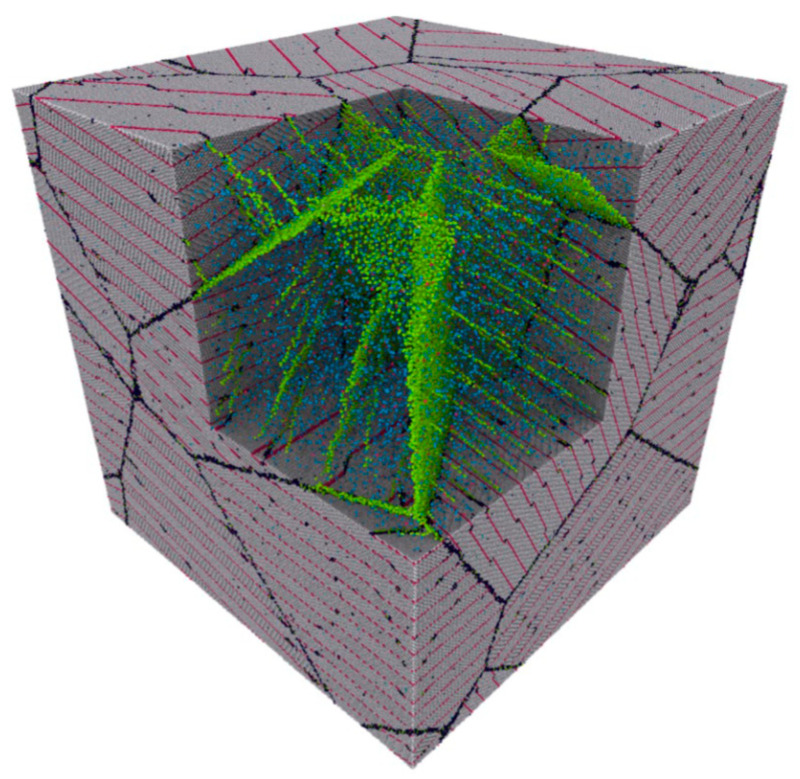
Simulated microstructure of Cu atoms doping Ag grain and twin boundaries; green represents Cu. Reproduced with permission from Reference [257]. Copyright 2019, Springer Nature.

For AgCu nanoalloys with nanograin microstructures, Ag atoms are doped into the grain boundaries of the Cu matrix, and vice versa. In the case of a doped Cu matrix [256], a hybrid Monte Carlo/molecular dynamics simulation showed that Ag atoms segregated in the grain boundaries between Cu crystals. Ag atoms gradually aggregated along grain boundaries as the Ag concentration was increased (Figure 13a,b). After exceeding the threshold (50 atoms/nm^2^), Ag atoms formed wetting nanolayers along the grain boundaries (Figure 13c–e). The reason is that these wetting nanolayers have lower energies [256]. In a similar fashion, when Cu atoms dope Ag, hybrid Monte Carlo/molecular dynamics simulations indicated that they segregated in grain or twin boundaries (Figure 14) [257].

### 6.3. Physicochemical Characterizations

It is clear that the NP surface plays an important role in its antibacterial behavior. For this reason, research on NP surface properties appears to be a key direction, particularly in determining their compositions, and how they might contribute to the antimicrobial behavior. Hence, physicochemical characterization approaches are commonly used, including TOF-SIMS, XPS, TEM, SEM, EDXS, XRD, nanoIR^®^ (Bruker, Billerica, MA, USA), etc.

#### 6.3.1. TOF-SIMS and XPS

Time-of-flight secondary ion mass spectrometry (TOF-SIMS) [258,259] is an appropriate characterization method for NP surface analysis. It detects fragments sputtered from the surface. It has a probe depth of ≤1 nm and can sensitively detect fractional layers of surface components. Hence, it is possible to use TOF-SIMS to determine which chemical groups exist on the surfaces of NPs, and which component is the most effective antimicrobial agent.

Another surface characterization technique, X-ray photoelectron spectroscopy (XPS) [260,261] can be used to determine chemical environments and oxidation states of elements on the surfaces of NPs. While less surface-sensitive than TOF-SIMS (it has a probe depth of 3–5 nm, depending on the kinetic energy of the emitted electron), it can detect whether Ag and Cu NPs have been oxidized, and which oxides have been produced.

**Figure 15:**
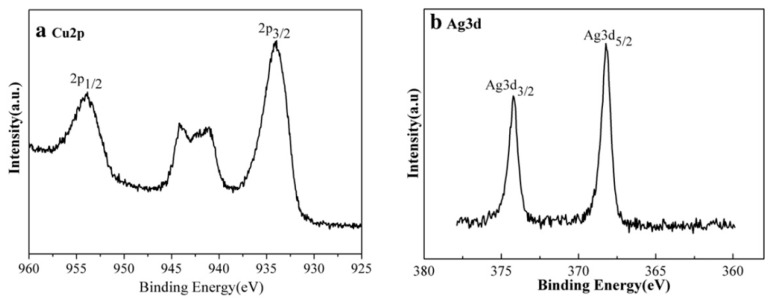
XPS spectra of (**a**) Cu2p and (**b**) Ag3d in AgCu_2_O nanoalloys. Reproduced with permission from Reference [242]. Copyright 2015, Elsevier.

Since XPS can detect element quantitatively, the surface composition of AgCu is approximately equal to the area ratio of Ag3d_5/2_ to Cu2p_3/2_ spectra, which are the most prominent spectral peaks of these elements [8]. XPS also indicated that, for both Ag and Cu NPs deposited onto the surface of polyurethane, Cu^0^ was found only at the surface of the AgCu nanofilm with an Ag:Cu ratio of 1:1, a ratio that exhibits the most efficient antimicrobial activity [116]. For AgCu_2_O nanoalloys (Figure 15), the major Cu2p_3/2_ peak, at ~934 eV, indicates the presence of Cu^+^, while the shake-up satellite indicates that some of it has been partially oxidized to CuO. The Ag3d_5/2_ peak, at ~368 eV, indicates the presence of Ag^0^ [242]. This indicates that Ag has formed a shell around partially oxidized Cu_2_O.

**Figure 16:**
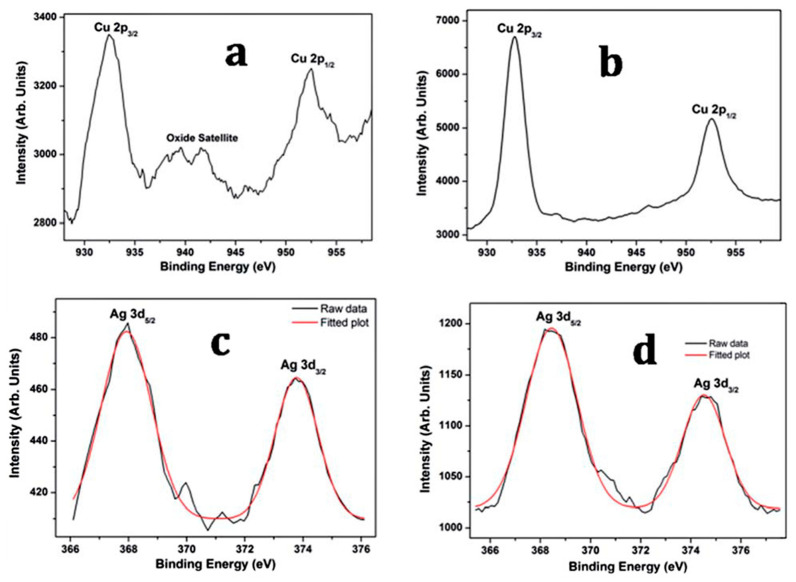
XPS spectra of Cu2p (**a**) before and (**b**) after sputtering, and Ag3d (**c**) before and (**d**) after sputtering, in Ag_shell_Cu_core_ nanoalloy. Reproduced with permission from Reference [262]. Copyright 2015, Royal Society of Chemistry.

Changing the XPS probe depth, by sputtering away the outer surface, can be used to identify the core-shell structures of nanoalloys. While the Cu shake-up satellite indicates the presence of CuO (Figure 16a), sputtering to a depth of 10 nm causes the satellite region to disappear (Figure 16b), which indicates the existence of CuO at the surface, rather than in the core. By contrast, Ag is not easily oxidized (Figure 16c). Moreover, the relative increase in the Cu:Ag ratio on sputtering reveals that, aside from CuO, Cu tends to occupy the core of the structure (Figure 16b,d) [262], as expected for a Ag_shell_Cu_2_O_core_ structure.

Generally, TOF-SIMS is combined with XPS to investigate the chemical components more accurately, with one supporting the results of the other [81,263,264]. In one report, N was detected by TOF-SIMS, but not by XPS, which means N-containing species, at very low concentrations, are confined on the surface of Ag NPs [86]. Thus, TOF-SIMS can not only confirm the XPS results, but often provide more detail.

#### 6.3.2. TEM and EDXS

**Figure 17:**
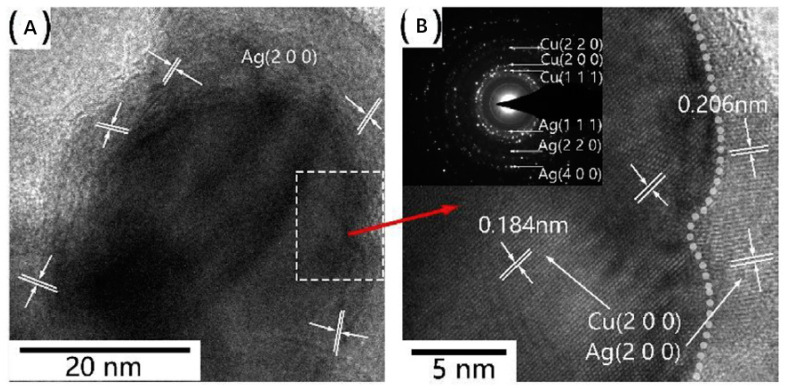
(**A**) HRTEM image of core-shell microstructure, in which Cu is the core, and Ag the shell; (**B**) magnified HRTEM image. Reproduced with permission from Reference [268]. Copyright 2017, Elsevier.

Transmission electron microscopy (TEM) [265] is frequently used to observe NP morphologies and antimicrobial activities; high-resolution transmission electron microscopy (HRTEM or HREM) is a particularly informative imaging mode. TEM offers images of internal information such as crystal structure, morphology, and mechanical stress, with resolutions down to 50 pm. It is generally used to evaluate morphological features, such as NP sizes and shapes [112], or to observe the antimicrobial behavior of NPs penetrating into bacterial cell [266]. NP surface modification [132], as well as substrate decoration [127,267], can also be directly detected by TEM. It has been reported that both Ag and Cu NPs can induce cell wall separation from cell membranes, leading to the further damage of cell walls and membranes and, ultimately, cytoplasmic material release, all of which have been observed by TEM [111]. HRTEM can even provide the details of the nanocrystalline structure (Figure 12). As another example, the core-shell microstructures of AgCu nanoalloys, with different orientations of the crystal facets of Ag (2, 0, 0) and Cu (2, 0, 0), can be clearly observed via HRTEM (Figure 17) [268].

**Figure 18:**
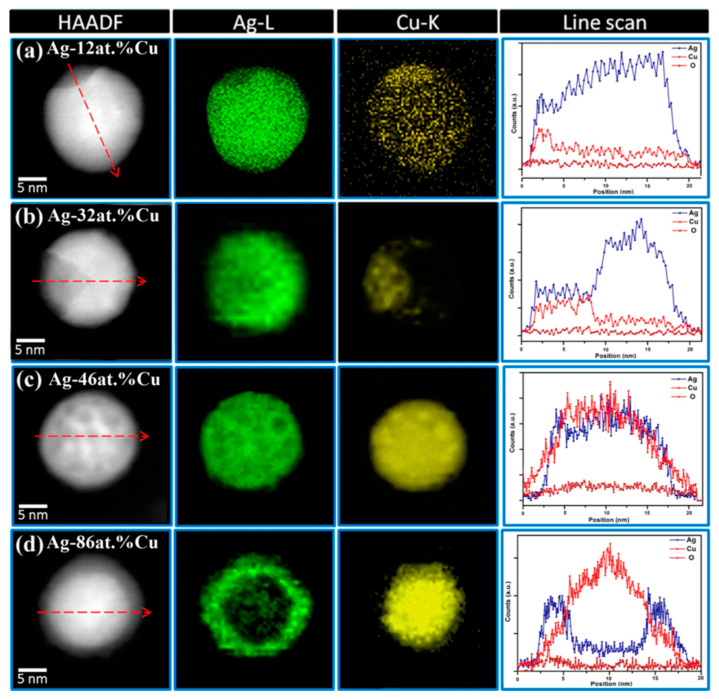
TEM-EDXS in high-angle annular dark field (HAADF) mode analysis of four different types of distribution of Ag and Cu in nanoalloy. Red arrow on HAADF image is the position and direction of line scan. Reproduced with permission from Reference [237]. Copyright 2014, American Chemical Society.

Energy-dispersive X-ray spectroscopy (EDS, EDX, EDXS or XEDS) [269] is a widely employed characterization technique, often used for elemental and chemical analyses. Commonly, EDXS is used with TEM, to obtain chemical proportions and distributions in NPs. TEM-EDXS spectra of AgCu nanoalloys provided the ratio of Ag and Cu, which was consistent with the initial ratio of Ag and Cu salt precursors [227]. Different microstructures, such as homogeneous nanoalloy and core-shell AgCu microstructures, can be distinguished by means of EDXS. EDXS chemical analysis indicated that Ag and Cu are well mixed in the AgCu nanoalloy, although the Cu content is higher in the shell and the Ag content in the core, of core-shell microstructure [190]. Another more comprehensive report of TEM-EDXS spectra, in the HAADF (high-angle annular dark field) mode, distinguished four different types of component distribution in AgCu nanoalloys (Figure 18) [237].

EDXS can also be used in combination with XPS: for a mixture of Ag and Cu NPs, XPS revealed that the Ag content was greater than that evaluated by EDXS. This was explained as follows: the act of mixing the Ag and Cu NPs formed a core-shell microstructure, with Ag at the surface [246].

#### 6.3.3. XRD

**Figure 19:**
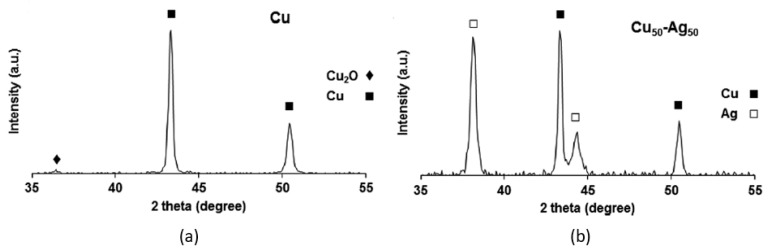
XRD spectra of (**a**) Cu NPs and (**b**) Cu_50_-Ag_50_ NPs. Reproduced with permission from Reference [246]. Copyright 2016, Royal Society of Chemistry.

**Figure 20:**
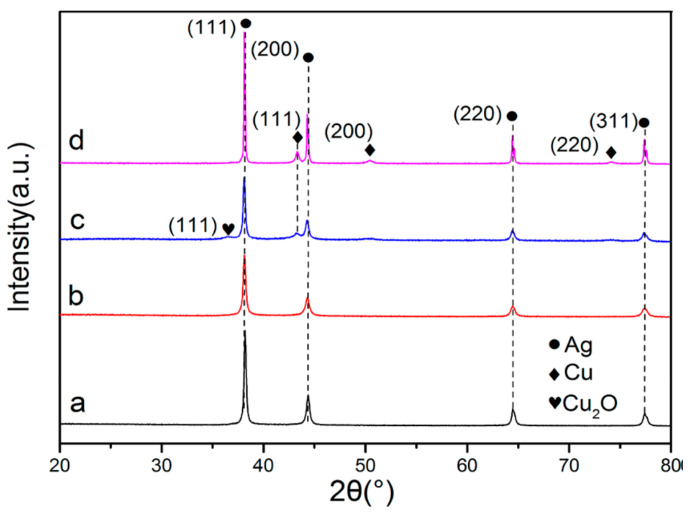
XRD spectra: (a) no Cu peaks in the spectrum of Ag_6_Cu; (b) no Cu peaks in the spectrum of Ag_2_Cu; (c) peaks of Cu and Cu_2_O in the spectrum of AgCu_3_; (d) peaks of Cu and Cu_2_O in the spectrum of AgCuO_x_. Reproduced with permission from Reference [272]. Copyright 2019, American Chemical Society.

X-ray diffraction (XRD) [270] is used to determine crystalline microstructure. When studying AgCu nanoalloys, it was used to determine whether the microstructure is a single phase or separated phases, through observing whether there is a shift of the diffraction peaks [242]. One study deduced that the microstructure was actually phase-separated instead of an alloy, because of the existence of distinct diffraction peaks of both Ag and Cu in mixed Ag–Cu NPs (Figure 19) [246]. A second study, on Cu_2_O-Ag nanocomposites, reached a similar conclusion [271]. If Ag and Cu formed a homogeneously distributed nanoalloy microstructure, the diffraction peaks of Cu and Cu oxides would not appear in the XRD (Figure 20a,b) [272]. On the contrary, the presence of the diffraction peaks of Cu and Cu oxides in the spectrum indicated that the phases are separated (Figure 20c,d) [272]. The formation of oxide-free AgCu nanoalloys can also be determined by XRD, through the absence of the CuO diffraction peak at 61.7° and the Cu_2_O diffraction peak at 37.5° [227].

#### 6.3.4. Other Characterization Techniques

NanoIR^®^ [273,274] is a new technique, which has a great potential to analyze NPs. Conventional IR technology provides information on functional groups, whereas atomic force microscopy (AFM) offers information on surface morphology. It is common to use both IR and AFM to evaluate NPs [29,117,132,154]. In contrast, nanoIR^®^ possesses these two functions simultaneously, although no research group appears to have yet used it to analyze NPs.

In addition to the characterization methods above, thermal analyses (i.e., TGA and DSC) [275], surface-enhanced Raman spectroscopy [276], and UV-vis spectroscopy [277] have also been used to study the antimicrobial behavior of NPs.

### 6.4. Antimicrobial Potential of Mixed Ag–Cu NPs and AgCu Nanoalloys

AgCu alloys, clad as millimeter-thick coating layers on stainless steel substrates, were found to exhibit high antimicrobial activity [278]. Unfortunately, the physicochemical and biological properties of mixed Ag–Cu NPs and AgCu nanoalloys are presently not clearly understood, even though their excellent antimicrobial activities are well known. A comparison is found, in Table 2, among Ag, Cu, mixed Ag–Cu and AgCu nanoalloy NPs. Because of different microstructures and Ag:Cu ratios, antimicrobial conclusions are not always identical.

Polyester fabrics, coated by mixed Ag–Cu NPs without specific microstructures, have shown significantly enhanced antimicrobial properties over Ag-treated fabrics [279]. Another example, which did not evaluate the AgCu microstructure, compared single Ag and Cu NPs, mixed Ag–Cu NPs, and AgCu nanoalloys, and concluded that the antimicrobial activity of AgCu nanoalloys is much greater than those of the other three [11]. Further, porous AgCu nanoalloys revealed much stronger antimicrobial activities to both Gram-positive and Gram-negative bacteria than either Ag or Cu NPs, when the Ag:Cu ratio was 1:1 [233]; changing the ratio decreased the activity. Another study found that a AgCu nanoalloy with the Ag:Cu ratio of 13:7 had the strongest antimicrobial activity, because it released the greatest amount of ions [245].

In the case of AgCu nanoalloys with core-shell microstructures, it was found that different Ag:Cu ratios demonstrated different antimicrobial efficacies, in which the mixture with a higher ratio (0.3) showed better antimicrobial activity than lower ratios (0, 0.1, 0.2) [152]. Similar conclusions was obtained in another study, which indicated that Ag and Cu NPs, in a core-shell microstructure, had excellent antimicrobial activities against both Gram-positive and Gram-negative bacteria, especially when the Ag:Cu ratio was 0.4 [246]. However, in the case of antifungal properties, Ag_core_Cu_shell_ nanoalloys were found not to demonstrate higher activity than single Ag NPs [231]. Another viewpoint holds that Ag_shell_Ag–Cu_core_ nanoalloys have long-term antimicrobial activity because the Ag shell has excellent oxidative stability [232]. Additionally, CuO_2_ NPs, combined with Ag NPs to form core-shell nanoalloys, exhibited higher antimicrobial action than did CuO_2_ NPs [241,242]. However, the antimicrobial properties of core-shell AgCu nanoalloys were still lower than those of uniformly distributed AgCu nanoalloys, which may stem from weaker Ag–Cu interactions in core-shell structures [22].

It was suggested that the much stronger antimicrobial activity of Ag–Cu nanoalloys is due to the greater amount of released Ag ions [280]. According to this study, charge transfer exists only at the interface of phase-separated Ag and Cu, thereby causing a weak release of Ag ions, whereas Ag atoms, surrounded by Cu atoms, can be oxidized, releasing more ions. In contrast, it was suggested that the higher antimicrobial properties of AgCu nanoalloys were due to the much larger (28×) amount of Cu ions released from nanoalloys than from single Cu NPs [271,281].

Another study proposed that the proteins and enzymes of microbes are susceptive to either Ag or Cu, and that AgCu nanoalloys can provide both metals, which exhibit synergistic antimicrobial activity [224]. However, as indicated in the probable antimicrobial mechanisms discussed above, the release of ions may be neither the correct, nor the only, reason. It appears necessary to determine the specific kinds of ROS generated: for example, titanate nanotubes, embedded with Ag and Cu NPs, showed much more effective antimicrobial activity than either Ag or Cu NPs, when under visible light radiation (Ag–Cu heterojunctions can reduce electron-hole recombination, and generate higher amounts of ROS, such as •O_2_^−^ and H_2_O_2_) [282].

## 7. Conclusions and Perspectives

While the data reported are inconsistent, due, at least in part, to our ignorance in framing the studies so as to take all the variables into consideration, two conclusions seem obvious: first, the operative antimicrobial mechanism depends on the conditions under which the study was carried out; second, the antimicrobial efficacy follows the order, Ag NPs ≈ Cu NPs < mixed Ag–Cu NPs < AgCu nanoalloys.

The possible mechanisms discussed herein imply that dry NPs, their ions, and the ROS produced, all exert some antimicrobial effect. It is, therefore, difficult to distinguish which is the predominant mechanism under any given set of circumstances. It will be important for future studies to concentrate on specifically controlled experiments, to determine explicit details, such as which kinds of ROS are produced, and how NPs kill microbes under dry conditions without releasing ions.

Further, there is a synergy in using mixed Ag–Cu NPs, indicating that the antimicrobial modes of Ag and Cu NPs differ. In addition, the fact that antimicrobial properties of mixed Ag–Cu NPs are weaker than those of AgCu nanoalloys tells us there is something present in the AgCu nanoalloy that does not exist in the simple Ag–Cu mixture. Future work should focus on this synergy, and the relationship between the surface structures of AgCu nanoalloys and their antimicrobial action.

## Figures and Tables

**Table 1:** Summary of implant surface modifications by Ag NPs, Cu NPs, Ag–Cu NPs mixture, and Ag–Cu nanoalloys.

Method	Substrate	NPs	Size (nm)	Coating Thickness (nm)	Application		Reference
Plasma immersion ion implantation (PIII)	Stainless steel	Ag NPs	5–16	Unknown	Implants		[198]
	Titanium	Ag NPs	Unknown	80	Implants		[80]
		Ag NPs	4–19	25	Implants		[209]
		Ag NPs	5–40	Unknown	Dental implants		[210]
		Ag NPs	5	50	Implants		[216]
	Polyethylene	Ag NPs, Cu NPs	Unknown	Unknown	Joint implants		[193]
Magnetron sputtering	Titanium dioxide	Ag NPs	14–42	Unknown	Coating		[90]
		Ag NPs	Unknown	24.6–73.8	Coating		[203]
	Tantalum nitride	Ag NPs, Cu NPs, Ag–Cu NPs mixture	Unknown	100	Coating		[196]
		Ag NPs	10–200	700	Coating		[217]
	Tantalum oxides	Ag NPs	<160	600–700	Coating		[218]
	Titanium-aluminum-nitride	AgCu nanoalloys	20–1100	100	Coating		[219]
	Polyether-ether-ketone	Ag NPs	Unknown	3–12	Implants		[202]
	Polyurethane	Ag–Cu NPs mixture	<5	Unknown	Catheter		[116]
		Ag–Cu NPs mixture	Unknown	80	Catheter		[170]
		Ag–Cu NPs mixture	Unknown	22	Catheter		[220]
Plasma electrolytic oxidation (PEO)	Titanium alloy	Ag NPs Cu NPs Ag–Cu NPs mixture	7–60	Unknown	Implants		[197]
		Ag NPs	37	Unknown	Implants		[212]
	Magnesium alloy	Cu NPs	Unknown	8000–11,000	implants		[200]
	Titanium	Cu NPs	Unknown	5000–10,000	Implants		[213]
						Reducing Agent	
Chemical reduction	Titanium dioxide	Ag NPs	102	Unknown	Coating	NaOH	[119]
		Ag NPs	600–1000	Unknown	Coating	Dehydrated ethanol	[205]
		Ag NPs	10–30	Unknown	Coating	Ammonia	[206]
		Ag NPs	20	Unknown	Coating	Glucose	[221]
	Titanium	Ag NPs	3–5	Unknown	Implants	UV&Methanol	[207]
		Ag NPs	30	Unknown	Implants	Ascorbic acid	[222]

**Table 2:** Summary of microstructures and antimicrobial properties of Ag–Cu combinations.

Microstructure	Size (nm)	Ratio (Ag:Cu)	Control NPs	Strain	Antimicrobial Efficacy	Reference
Unknown	200	1:1	Ag NPs Cu NPs Ag–Cu NPs	*E. coli* *B. subtilis*	AgCu > Ag > (Ag–Cu) > Cu	[11]
	30–55	3:1 1:1 1:3	Ag NPs Cu NPs	*E. coli* *S. aureus*	Cu > AgCu_3_ > AgCu > Ag_3_Cu > Ag	[126]
	2–5	1:1 2:1 3:1	Ag NPs Cu NPs	*E. coli*	AgCu > Ag_2_Cu > Ag_3_Cu > Cu > Ag	[116]
	20–30	1:1	Ag NPs	*E. coli* *K. pneumoniae* *E. aerogenes* *P. mirabilis* *P. aeruginosa* *S. aureus*	AgCu > Ag	[188]
				*E. faecalis*	Ag > AgCu	
	7–60	1:1 3:1	Ag NPs Cu NPs	*S. aureus*	AgCu ≈ Ag_3_Cu > Ag > Cu	[197]
	20	1:1 3:1	Ag NPs Cu NPs	*E. coli*	AgCu > Ag_3_Cu > Ag ≈ Cu	[233]
				*S. aureus*	AgCu> Ag_3_Cu ≈ Ag ≈ Cu	
	215–788	1:1	Ag NPs Cu NPs	*S. aureus*	AgCu ≈ Cu > Ag	[279]
				*K. pneumoniae*	AgCu > Cu > Ag	
	Unknown	3:1 1:1 1:3	Ag NPs Cu NPs	*S. aureus*	Ag_3_Cu > AgCu > Ag > AgCu_3_ > Cu	[282]
	30–80	Unknown	Ag NPs Cu NPs	*E. coli*	AgCu > Ag or Cu	[281]
				*P. aeruginosa*		
				*S. typhi*		
	20–180	0.1:3 0.2:2 0.3:1	Ag NPs Cu NPs	*E. coli*	Ag_0.2_Cu_2_ ≈ Ag_0.3_Cu > Ag_0.1_Cu_3_ > Ag > Cu	[283]
				*S. aureus*	Ag_0.2_Cu_2_ is the best	
	3	1:1	Ag NPs Cu NPs	*E. coli*	AgCu > Ag > Cu	[284]
	500–600	Unknown	Cu NPs	*E. coli*	AgCu > Cu	[285]
				*S. aureus*		
Core-shell (Ag_Core_Cu_shell_)	20–70	1:1	Ag NPs Cu NPs Cu_Core_Ag_shell_ NPs	*E. coli*	Ag_core_Cu_shell_ > Cu_core_Ag_shell_ > Ag > Cu	[231]
				*K. pneumoniae*	Ag_core_Cu_shell_ > Cu_core_Ag_shell_ > Ag > Cu	
				*P. aeruginosa*	Ag_core_Cu_shell_ > Cu_core_Ag_shell_ ≈ Ag > Cu	
				*S. aureus*	Ag_core_Cu_shell_ > Cu_core_Ag_shell_ > Ag > Cu	
				*A. fumigatus*	Cu_core_Ag_shell_ > Ag > Ag_core_Cu_shell_	
	<100	22:78 65:35 94:6	Ag NPs Cu NPs	*E. coli*	Ag_65_Cu_35_ ≈ Ag_22_Cu_78_ > Ag_94_Cu_6_ > Ag ≈ Cu	[245]
				*S. aureus*	Ag_65_Cu_35_ ≈ Ag_22_Cu_78_ > Ag_94_Cu_6_ > Ag > Cu	
Core-shell (Cu_Core_Ag_shell_)	1000–1500	1:10 1:5 3:10	Cu NPs	*E. coli*	Ag_3_Cu_10_ > AgCu_5_ > AgCu_10_ ≈ Cu	[152]
				*S. aureus*	Ag_3_Cu_10_ > AgCu_5_ > AgCu_10_ > Cu	
	7	1:3 2:3 1:1 4:1	Cu NPs	*E. coli*	Ag_4_Cu > AgCu > Ag_2_Cu_3_ ≈ AgCu_3_ ≈ Cu	[246]
				*S. aureus*	Ag_4_Cu ≈ AgCu ≈ Ag_2_Cu_3_ ≈ Cu > AgCu_3_	
				*B. subtilis*	AgCu > Ag > (Ag–Cu) > Cu	
	150	Unknown	Cu_2_O NPs	*E. coli*	Cu_2_OAg > Cu_2_O	[242]
	400–500	Unknown	Cu_2_O NPs	*S. aureus*	Cu_2_OAg > Cu_2_O	[271]
				*P. aeruginosa*		
	3000	Unknown	μCuO Ag NPs	*E. coli*	μCuOAg > Ag > μCuO	[241]
				*Salmonella*	μCuOAg > Ag > μCuO	
				*Listeria*	CuO > CuO > Ag	
	10–30	1:1 3:1 5:1	-	*E. coli*	AgCu ≈ Ag_3_Cu ≈ Ag_5_Cu	[286]
				*S. aureus*	Ag_5_Cu > Ag_3_Cu > AgCu	
				*A. flavus*	Ag_5_Cu > Ag_3_Cu > AgCu	
				*C. albicans*	Ag_5_Cu > Ag_3_Cu > AgCu	
Nanoalloy	35–50	Unknown	Ag NPs Cu NPs Ag_Core_Cu_shell_ NPs Cu_Core_Ag_shell_ NPs	*C. albicans*	Homogeneous AgCu > Ag_core_Cu_shell_ > Ag> Cu_core_Ag_shell_ > Cu	[22]
				*E. coli*	Homogeneous AgCu > Cu_core_Ag_shell_ > Cu	
				*S. aureus*	Homogeneous AgCu > Cu > Cu_core_-Ag_shell_	
	5–7	1:1	Ag NPs Cu NPs	*E. coli* *S. aureus*	AgCu > Cu > Ag	[224]
	2.1	1:1	Ag NPs Cu NPs biphase AgCu	*E. coli*	Homogeneous AgCu > phase-separated AgCu ≈ Ag > Cu	[280]
				*S. aureus*	Homogeneous AgCu >Ag > biphase AgCu > Cu	

## Data Availability

No new data were created or analyzed in this study. Data sharing is not applicable to this article.

## Appendix A

**Table A1:** Data examined in the Polytechnique Montreal library Compendex database.

Objective	Key Words
Cu	(antimicrob* OR antibact* OR biocid* OR antibiof* OR antivir* OR antifung*) AND (copper* OR Cu OR CuO OR CuNP* OR nanocopper OR nano-copper OR nanoCu OR Cu-*)
Ag	(antimicrob* OR antibact* OR biocid* OR antibiof* OR antivir* OR antifung*) AND (silver OR Ag OR AgNP* OR nanosilver OR nano-Ag OR nanoAg OR Ag-*)
Cu nano	(antimicrob* OR antibact* OR biocid* OR antibiof* OR antivir* OR antifung*) AND (copper nano* OR Cu NP* OR CuNP* OR Cu nano*)
Ag nano	(antimicrob* OR antibact* OR biocid* OR antibiof* OR antivir* OR antifung*) AND (silver nano* OR Ag NP* OR AgNP* OR Ag nano*)
Combination of Ag, Cu nano	(antimicrob* OR antibact* OR biocid* OR antibiof* OR antivir* OR antifung*) AND (silver-copper OR copper-silver OR Ag-Cu OR Cu-Ag OR silver/copper OR copper/silver OR Ag/Cu OR Cu/Ag OR Ag@Cu OR Cu@Ag OR AgCu OR CuAg) AND (nano* OR alloyed nano* OR nanoalloy* OR nano-alloy*)

* refers to the end of the syllables researched.
